# Accuracy of continuous glucose monitoring in critically ill patients

**DOI:** 10.1016/j.ccrj.2025.100118

**Published:** 2025-10-17

**Authors:** John D. Santamaria, Ebony Selers, David Reid

**Affiliations:** a St Vincent's Hospital Melbourne, 41 Victoria Parade, Fitzroy, VIC 3065, Australia; b Royal Melbourne Hospital, 300 Grattan Street, Parkville VIC 3052, Australia

**Keywords:** Human, Intensive care units, Continuous glucose monitoring, Glucose/analysis, Insulin infusions

## Abstract

**Objective:**

Hyperglycaemia requiring insulin infusions is common among critically ill patients. Attempts to tightly control glucose levels in the intensive care unit (ICU) have had mixed results partly due to hypoglycaemia. Continuous glucose monitoring (CGM) has been widely adopted among ambulatory persons with diabetes but tested only on small numbers of patients in the ICU. This study was undertaken to address the accuracy of CGM in a group of critically ill patients.

**Setting and outcomes:**

This observational study included critically ill ICU patients admitted between 2019 and 2023 to a tertiary referral, university-affiliated hospital with a single 19-bed ICU. Patients requiring insulin to control hyperglycaemia had a sensor (Dexcom G4 or G6) attached. Accuracy was assessed as bias and mean absolute relative difference (MARD). Results are presented as median (interquartile range).

**Results:**

103 patients were assessed. The majority (97%) were mechanically ventilated and on vasopressors (92%), and a quarter required renal replacement therapy. Men comprised 68% of the cohort; median age was 63 years, and body mass index was 29.8. Sensors were applied for 79.5 (48–160) hours. The median bias was −0.30 mmol/L (−0.55, 0.05), and MARD was 11.23 (8.10–15.03). No clinical hypoglycaemia occurred with sensors in place.

**Conclusion:**

In 103 patients with multiorgan failure on insulin for hyperglycaemia, CGM had acceptable bias and MARD and no hypoglycaemia. Given the accuracy seen in this trial, CGM could be considered in future trials of glucose management and used as a safety measure to reduce hypoglycaemic events.

**Cinical trials registration:**

The study was not registered with the Australian and New Zealand Clinical Trials Registry).

## 1 Introduction

There is consistent evidence that good control of glucose levels, as gauged by glycated haemoglobin levels, in ambulatory people with diabetes leads to less long-term complications.[1], [2], [3] For hospitalised patients with and without diabetes, dysglycaemia adversely influences outcomes, leading to the proposal that blood glucose levels should be considered as the “fifth vital sign”.^4^ However, optimal glucose targets for inpatients with or without diabetes remain uncertain. Tight glucose control in the intensive care setting (4.0–6.5 mmol/L) was initially shown to improve mortality^5^^,^^6^ but was not replicated in a larger multicentre trial.^7^ More recently, higher glucose targets for critically ill patients with type 2 diabetes decreased hypoglycaemic events but possibly resulted in more complications.^8^

At the same time, there has been discussion around how glycaemic control in the intensive care unit (ICU) should be assessed and what constitutes good glycaemic control. Three measures of glycaemic control have emerged, and each has been associated with clinical outcomes—mean glucose, variability of glucose, and hypoglycaemic events.^9^ It is possible that concentrating on one element of glycaemic control (e.g., mean glucose) could lead to worse outcomes through greater variability and more episodes of hypoglycaemia.

Continuous glucose monitoring (CGM) using subcutaneous sensors has become increasingly popular in the management of ambulatory patients with type 1 diabetes and has had positive impacts on glycaemic control and quality of life.^10^ These devices are simple to insert and provide measurements every 2–5 min. Such feedback has led to more time spent in recommended glucose ranges^11^ without increasing episodes of hypoglycaemia.^12^^,^^13^ Although we still await long-term studies on mortality and morbidity, some of these systems are robust and sufficiently safe to permit control of subcutaneous insulin infusion pumps thereby closing the loop on ambulatory blood glucose management.^14^

ICUs traditionally monitor glucose levels with either point-of-care capillary techniques or by glucose measured by blood gas analysers both of which provide good accuracy. However, these are done intermittently or at best hourly. This potentially puts patients receiving insulin infusions at a risk of hypoglycaemia between measures.

Use of current-generation continuous tissue glucose monitoring of critically ill patients has been reported on several occasions and in limited number of patients.[15], [16], [17], [18], [19], [20], [21], [22], [23], [24], [25], [26] The use of these newer devices was driven partly by the Coronavirus Disease (COVID) pandemic where strict isolation of patients sometimes led to reduced blood or capillary analysis. In these studies, patients had a limited number of medical conditions, yet CGM was effective and well embraced by clinical staff. However, concerns regarding the accuracy of CGM due to the concept of “lag time” and the difference between blood and tissue glucose levels, especially in the setting of rapidly changing blood glucose levels and reduced tissue perfusion, have been raised.^27^

In this prospective study, we investigated whether continuous tissue glucose monitoring using current-generation devices intended for ambulatory patients would provide good accuracy when compared to blood glucose measurements in a mixed cohort of intensive care patients requiring insulin infusions.

## 2 Methods

The study was undertaken at St Vincent's Hospital Melbourne which is a 450-bed tertiary university-affiliated hospital, which undertakes cardiac surgery and neurosurgery. Major organ transplantation, trauma, and burn patients are managed at other centres. There is a single ICU of 19 beds; 1600 patients are admitted each year; 13% for emergency neurosurgery and 31% for cardiac surgery. The study was approved by the quality assurance committee of the hospital (Approval QA 02/19, 14 January 2019); the need for informed consent was waived. The study commenced in 2019 but was placed on hold during 2020–2022 due to the COVID-19 pandemic and then completed in 2023.

Patients receiving insulin infusions to maintain blood glucose levels were eligible for enrolment with selection based on clinician and sensor availability. Our choice of devices was limited to those which connected to Bluetooth receivers every 3–5 min and were capable of setting alarms; this excluded the flash devices that required staff to directly attend the patient. The Dexcom G4 system was used initially followed by the Dexcom G6 system (Australasian Medical and Scientific, Sydney, Australia). Both systems comprise a sensor which, when applied to the skin (usually the outer upper arm), implants a small subcutaneous filament which measures tissue glucose. A small transmitter is attached to the sensor, and data are read by a receiver at the nurse's workstation at the end of the bed. Data are transmitted from the sensor to the receiver every 5 min. Additionally, each time blood glucose was measured (ABL90 Flex, Radiometer Pacific, Mt Waverley, Australia), that value was entered into the receiver. Tissue glucose readings and blood glucose readings were timestamped and identified within the receiver. This had a dual purpose. First, it provided a storage of data that could be downloaded for analysis and second, it allowed the algorithms within the devices to calibrate the tissue sensors for more accurate readings. Regular calibrations were required for the G4 system (initial and 12 hourly) but not necessarily for the G6 system. However, to ensure the proper timestamping of blood and tissue readings, the G6 was run in the “no-code” mode, and the algorithm was updated with each blood glucose measure.

An insulin infusion was commenced when the patient's blood glucose consistently exceeded 10 mmol/L, and infusion rates were modified according to the local protocol (e-Fig. 1, Online Supplement). Staff members were allowed but not encouraged to alter insulin infusion rates based on tissue glucose readings alone. The default alarms of the receivers remained active.

On completion of each patient study, data within the receivers were downloaded and analysed. A sample download is shown in e-Table 1 of the online supplement. The tissue glucose reading immediately (<2 min) before the blood glucose measurement was used to assess accuracy. The median number (interquartile range [IQR]) of blood glucose measurements entered within the receiver was 13 (8–20) per patient.

We used Bland–Altman measures of bias and precision^28^ as well as the mean absolute relative difference (MARD) and the Lin concordance correlation coefficient^29^ to define accuracy, as previously suggested.^30^ Glycaemic control included mean, standard deviation (SD) and coefficient of variation of glucose levels, and the number of hypoglycaemic events.^9^

Demographic data, diagnoses, therapies, comorbidities (Charlson^31^ and Elixhauser^32^), Hospital Frailty Risk Scores (HFRSs),^33^ and severity of illness scores (Acute Physiology and Chronic Health Evaluation [APACHE] II^34^ and Sequential Organ Failure Assessment [SOFA]^35^) were extracted from unit-based databases.

Data are expressed as median (IQR) for continuous variables and as number (%) for qualitative variables. Comparisons were made using Mann–Whitney U or Kruskal–Wallis tests for continuous variables and the Fisher's exact test for categorical variables. No sample size calculations were performed as this was a pilot study. Analyses were performed using Stata V18 (StataCorp, College Station, TX).

## 3 Results

We studied 103 patients. Patient characteristics are shown in Table 1; stratification by diabetic status is shown in e-Table 2 of the online supplement. Men comprised 68% of the cohort, median age was 63 (IQR: 55–69) years, and median body mass index was 29.8 kg/m^2^. Approximately half of the cohort was admitted from the operating room. The patients were moderately unwell with a median APACHE II score of 20 and SOFA score on day 1 of 10. Twenty-seven percent died before hospital discharge.

**Table 1:** Demographic features and characteristics of the patient cohort.

Number	103
Male	72 (69.9)
Age, years	63 (55–69)
Height, meters	1.73 (1.33–1.80)
Weight, kg	88 (79.0–109.7)
Body mass index (kg/m^2^)	29.8 (26.3–35.8)
ICU origin	
Emergency department	15 (14.6)
Operating room	50 (48.5)
General ward	11 (10.7)
Inter hospital transfer	27 (26.2)
Death in the ICU	17 (16.5)
Death in hospital	30 (29.4)
Comorbidities	
Diabetes	60 (58.2)
Hypertension	55 (53.4)
Ischaemic heart disease	35 (34.0)
Chronic lung disease	24 (23.3)
Current smoker	13 (12.6)
Severity of illness scores	
Acute physiology score	15 (11–21)
APACHE II score	20 (15–25)
Maximum SOFA score, first 24 h	10 (7–12)
Therapies	
Mechanical ventilation	100 (97.1)
Haemofiltration	26 (25.2)
Enteral feeding	84 (81.6)
Parenteral nutrition	12 (11.6)
Vasopressors	95 (92.2)
Renal function	
Creatinine (mmol/L)	0.10 (0.07–0.14)
eGFR (mL/min/1.73 m^2^)	62.2 (43.5–98.3)
eGFR <60	49 (47.6)
Pathology	
Bilirubin >20 mmol/L	36 (35.0)
Base deficit < −5.0	63 (61.2)
Highest glucose day 1, mmol/L	14.2 (12.4–16.4)
Lowest glucose day 1, mmol/L	6.2 (5.4–8.1)
Mean glucose day 1, mmol/L	10.4 (9.3–12.2)

Results are presented as median (IQR) for continuous variable and n (%) for categorical variables.

APACHE: Acute Physiology and Chronic Health Evaluation; ICU: intensive care unit; IQR: interquartile range; SOFA: Sequential Organ Failure Assessment.

Most patients required mechanical ventilation, a quarter needed renal replacement therapy, and nearly all required vasopressors (norepinephrine) during their stay.

A full list of diagnoses is shown e-Table 3 of the online supplement. Common diagnoses were cardiac surgery (coronary bypass, cardiac valve repair/replacement), cardiac arrest, sepsis, and intracerebral haemorrhage.

Fifty-seven percent had known diabetes. Other pre-existing conditions were hypertension (52%), ischaemic heart disease (33%), chronic lung disease (23%), and current smoking (14%). Charlson, Elixhauser, and HFRSs are shown in e-Table 4 of the online supplement; 32% of patients had two or more Charlson comorbidities, and 33% had an HFRS greater than 5.

Of the 103 patients, the Dexcom G4 was applied in 57 and Dexcom G6 in 46. Sensor details are shown in Table 2. The median (IQR) duration of sensor application was 79.5 (48–160) hours. During that time, the mean glucose was 7.8 mmol/L (SD: 2.05). The median (IQR) insulin infusion rate was 4.3 (3.2–5.9) units/hour.

**Table 2:** Sensor and glucose details for the 103 patients.

Sensor type (n,%)	
Dexcom G4	57 (55.3)
Dexcom G6	46 (46.7)
Sensor duration, hours	79.5 (48–160)
Glucose mean on sensor	7.83 (6.58–8.78)
Glucose standard deviation (SD) on sensor	2.05 (1.54–2.78)
Glucose coefficient variation on sensor	23.1 (19.4–28.8)
Insulin infusion rate, units/hr	
On sensor	4.3 (3.2–5.9)
Off sensor	4.4 (3.0–6.1)
Sensor readings per patient	943 (561–1865)
Bias (tissue minus blood glucose)	
G4	−0.32 (−0.55 to −0.14)
G6	−0.13 (−0.54 to +0.14)
Overall	−0.30 (−0.55 to +0.05)
Precision	
G4	1.66 (1.29–2.05)
G6	1.23 (1.03–1.51)
Overall	1.44 (1.10–1.89)
MARD∗	
G4	13.78 (8.70–16.35)
G6	9.90 (7.63–12.58)
Overall	11.23 (8.10–15.03)
Lin concordance correlation	
G4	0.727 (SD: 0.017)
G6	0.784 (SD: 0.017)
Number of low tissue glucose	
< 2.5 mmol/L	23
2.5–4.0 mmol/L	632
Number of low blood glucose	
< 2.5 mmol/L	0
2.5–4.0 mmol/L	4

Results are median (IQR) for continuous variables apart from Lin concordance correlation (mean/SD) and n (%) for categorical variables. See methodology for definitions of bias, MARD and calibration.

IQR: interquartile range; MARD: mean absolute relative difference.

Of the 104,220 readings recorded within the receiver, 1.2% readings were identified as blood gas results. The bias (CGM tissue glucose minus blood/capillary glucose) was −0.32 mmol/L for the G4 and -0.13 for the G6 (overall: −0.30, IQR: -0.55-0.05) indicating that tissue glucose slightly underestimated blood glucose. The Bland–Altman plots are shown in e-Figs 2,3 of the online supplement. The bias appeared stable across the range of glucose measurements. The MARD was 13.78 for the G4 sensor and 9.90 for the G6 sensor (overall: 11.23, IQR: 8.10–15.03).

Tissue and blood glucose measurements <4.0 mmol/L were rare. While there were more tissue measurements in the 2.5–4.0 range, there were only four blood glucose measurements in this range, and none required glucose administration. Time in range (4–10 mmol/L) was 68.9% even though this was not specifically targeted by bedside staff.

Biases were similar for patients with known diabetes compared to patients without known diabetes, level of renal impairment, duration of sensor application, and severity of illness scores (APACHE II and SOFA). (Table 3).

**Table 3:** Comparison of bias for different subgroups.

Group	Bias	p value
Diabetes pre-existing		
Diabetic	−0.31 (−0.62, −0.02)	0.322
Non-diabetic	−0.23 (−0.52, 0.08)	
Vasopressors required		
No	−0.16 (−0.27, 0.14)	0.216
Yes	−0.31 (−0.57, 0.36)	
eGFR group a		
<15	−0.16 (−0.63, 0.25)	0.420
15–30	−0.38 (−0.87, −0.11)	
31–60	−0.35 (−0.56, 0.08)	
>60	−0.19 (−0.53, 0.07)	
High vs low glucose^b^		
Low (<10 mmol/L)	−0.28 (−0.54, 0.07)	0.508
High (≥10 mmol/L)	−0.25 (−0.72, −0.09)	
Sensor duration		
≤ 80 h	−0.31 (−0.87, 0.06)	0.600
> 80 h	−0.24 (−0.52, 0.02)	
Primary diagnosis		
Medical	−0.25 (−0.54, 0.05)	0.736
Surgical	−0.32 (−0.56, 0.04)	
APACHE II score		
≤20	−0.18 (−0.46, 00.06)	
>20	−0.35 (−0.63, −0.05)	0.108

APACHE: Acute Physiology and Chronic Health Evaluation.

a Estimated glomerular filtration rate (mL/min/1.73 m^2^).

b Glucose based on mean glucose for whole admission.

## 4 Discussion

### 4.1 Main findings

In this pragmatic study of 103 critically ill patients receiving insulin for hyperglycaemia, we found that tissue glucose measurements had a bias of −0.30 mmol/L. The MARD was 13.78 for the G4 sensor and 9.90 for the G6 sensor for an overall MARD of 11.23. Although CGM was not intended to be used to adjust insulin infusion rates, we found that the time in range (4–10 mmol/L) was 68.9% and that the number of hypoglycaemic events was very low (0.61% of all readings for glucose less than 4.0 mmol/L).

### 4.2 Comparison to previous studies

Continuous tissue glucose monitoring (CGM) has become widespread in ambulatory people with diabetes, particularly in those with type 1 or insulin-dependent diabetes. Multiple studies of CGM in the aforementioned populations, especially when associated with alerts, have shown decreases of glycated haemoglobin, increases in time-within-target glucose range, and decreases in frequency of severe hypoglycaemic events when compared to self-monitored point-of-care glucose testing.[36], [37], [38]

The intensive care literature regarding the use of CGM has been less. In 2017, van Steen et al. undertook an extensive review of CGM systems in critically ill patients. Their review of studies reported between 2006 and 2016 included subcutaneous, intravascular, and transdermal systems. MARD varied between 5 and 23% and bias from −10.8 to +6.4 with better accuracy noted with intravascular devices. The studies also showed a reduction in hypoglycaemic events. However, the more modern devices such as Dexcom and Medtronic had not been released, and most of the devices reviewed are no longer in production. The COVID pandemic provided opportunities for more CGM studies. Sadhu et al. had 11 patients, six of whom wore the Dexcom G6 sensor, and they reported a bias of −1.94 mg/dL (0.11 mmol/L).^17^ In this study, the MARD was 11.1%. In a similar study, Agrawal used G6 sensors in 11 COVID-infected patients with most on insulin, vasopressors, and mechanical ventilation, with half receiving renal replacement therapy^18^; the MARD was 12.6%. The sensors were on for 9 days, and time in range was 46%. Later, a study in 30 patients with severe COVID infection had a time in range of 72% (SD: 16%) after 2 days of sensor application.^21^ Bias did not seem to vary with time in another study of 12 patients in a medical ICU.^19^ Friman et al.^24^ studied 40 ICU patients with the G6 sensor and found a MARD of 12.7%, while Voglova et al.^25^ had 61 critically ill post-transplant patients with G6 sensors that were calibrated initially 6-hourly for 2 days and then daily; they found a MARD of 9.4% but did note that accuracy might have been affected with high doses of ascorbic acid. A feature of these studies was the avoidance of hypoglycaemia with sensors applied. Some studies showed a reduction in blood gas or capillary sampling.

### 4.3 Implications

The appropriate targets for blood glucose in critically ill patients remain uncertain and based on evidence of low to moderate certainty.^39^ Tight glucose control studies targeting glucose levels between 4.5 and 6.0 mmol/L have reported varying outcomes on survival,[5], [6], [7]^,^^40^ and a recent meta-analysis concluded that intensive glucose control was not associated with reduced mortality but did increase the risk of hypoglycemia.^41^ In a recent study, type 2 diabetic patients admitted to the ICU and randomised to a target of 10–14 mmol/L ended up having greater 90-day mortality than those randomised to a standard target of 6–10 mmol/L (29.5% vs 24.9%).^8^

Mean glucose levels were used to assess the effectiveness of control in these studies, but it is known that glucose variability and hypoglycaemia are independently associated with mortality in critically ill patients.^9^ The incidences of patients experiencing severe hypoglycaemia (<2.2 mmol/L) in the four studies of tight glucose control vary between 1.0% and 16.8% (see eTable 5). The importance of hypoglycaemia was highlighted in a follow-up analysis of the NICE-SUGAR study.^42^ Looking at 90-day mortality, these authors found a mortality of 23.5% for no hypoglycaemia, 28.5% for patients who experienced moderate hypoglycaemia (2.3–3.9 mmol/L), and 28.5% for those with severe hypoglycaemia (≤2.2 mmol/L). The higher glucose target study^8^ reported hypoglycaemia (<4 mmol/L) in 4.7% allocated to the higher target compared to 18.2% in the lower target.

Given the uncertainties raised by these studies, the most recent guidelines from the Society of Critical Care Medicine have recommended glucose targets between 6.1 and 10.0 mmol/L with frequent monitoring (≤1 hourly) of blood glucose to avoid hypoglycaemia.^39^ Continuous tissue glucose monitoring could also provide a relatively inexpensive option by providing glucose estimates frequently (5-minutely) and alarms to warn of actual or impending hypoglycaemia as well as helping to maintain glucose within target ranges and possibly reducing variability. Our study suggests that current-generation CGMs have sufficient accuracy to fulfil this role.

### 4.4 Strengths and limitations

Our study of 103 patients is one of the largest to date and comprised patients with a variety of diagnoses. The patients were all critically unwell with elevated severity of illness scores and high rates of ventilation, vasopressors, and renal replacement therapy while receiving insulin infusions. They also had a diverse range of primary conditions. The study used modern or next-generation technology to monitor tissue glucose levels resulting in low bias and precision, and finally, the bedside nurse managed the insulin infusions.

There are some limitations of the study. First, it was performed at a single tertiary centre ICU and patients with trauma and major organ transplantation were not included.

Second, bedside staff members were not instructed to use the tissue glucose measurements to change insulin infusion rates. However, the CGM readings were freely visible on the receiver display and results were entered into the medical record each hour. We did not silence alarms for high or low tissue glucose readings, so it was likely that knowledge of tissue glucose prompted staff to verify results with capillary or blood glucose estimations and/or to adjust rates of insulin infusions.

Third, we did not adjust for the use of paracetamol/acetaminophen which was being administered to the majority of patients. In contrast to earlier models, the G6 sensor has a semipermeable membrane which blocks diffusion of the drug to the sensor so that any possible interaction would be minimised.^43^ As noted earlier, with high-dose ascorbic acid, there may be other medications that could potentially interact with sensors. This highlights the importance of still estimating blood glucose levels (especially when tissue readings are very low or high), but such tests need not be done frequently.

Fourth, there is a cost to tissue glucose monitoring. The single-use sensor costs $40 and can be used for 7–10 days. The transmitter was not a single-use one costing $500 and lasts for 3 months, and the receiver was a one-off cost of $500. These costs are additional but relatively small when compared to the estimated daily cost of an ICU bed of $4375^44^ and the extended length of stay of those who have hypoglycaemia.^45^

## 5 Summary

This pilot study of 103 critically ill patients has shown that continuous tissue glucose monitoring compares favourably with traditional measures of blood glucose. The bias (CGM vs conventional) was affected by neither severity of illness nor therapies administered to treat these patients. It would seem an appropriate time to evaluate CGM in trials of glucose management where time in range and avoidance of hypoglycaemia are of prime importance.

## Declaration of Generative AI and AI-assisted technologies in the writing process

During the preparation of this work, the authors have not used any AI or AI-assisted technologies.

## Funding

This research did not receive any specific grant from funding agencies in the public, commercial, or not-for-profit sectors.

## Conflict of interest

The authors declare that they have no known competing financial interests or personal relationships that could have appeared to influence the work reported in this paper.

## References

[1] The Diabetes Control and Complications Trial (DCCT) (1993). The effect of intensive treatment of diabetes on the development and progression of long-term complications in insulin-dependent diabetes mellitus. N Engl J Med.

[2] The Diabetes Control and Complications Trial (DCCT) (1995). The relationship of glycemic exposure (HbA1c) to the risk of development and progression of retinopathy in the diabetes control and complications trial. Diabetes.

[3] UK Prospective Diabetes Study (UKPDS) Group (1998). Intensive blood-glucose control with sulphonylureas or insulin compared with conventional treatment and risk of complications in patients with type 2 diabetes (UKPDS 33). UK prospective diabetes study (UKPDS) group. Lancet.

[4] Kesavadev J., Misra A., Saboo B., Aravind S.R., Hussain A., Czupryniaket L. (2021). Blood glucose levels should be considered as a new vital sign indicative of prognosis during hospitalization. Diabetes Metab Syndr.

[5] van den Berghe G., Wouters P., Weekers F., Verwaest C., Bruyinckx F., Schetz M. (2001). Intensive insulin therapy in critically ill patients. N Engl J Med.

[6] Van den Berghe G., Wilmer A., Hermans G., Meersseman W., Wouters P.J., Milants I. (2006). Intensive insulin therapy in the medical ICU. N Engl J Med.

[7] The NICE-SUGAR Study Investigators (2009). Intensive versus conventional glucose control in critically ill patients. N Engl J Med.

[8] Poole A.P., Finnis M.E., Anstey J., Bellomo R., Bihari S., Birader V. (2022). The effect of a liberal approach to glucose control in critically ill patients with type 2 diabetes: a multicenter, parallel-group, open-label randomized clinical trial. Am J Respir Crit Care Med.

[9] Mackenzie I.M., Whitehouse T., Nightingale P.G. (2011). The metrics of glycaemic control in critical care. Intensive Care Med.

[10] Polonsky W.H., Hessler D., Ruedy K.J., Beck R.W., Group D.S. (2017). The impact of continuous glucose monitoring on markers of quality of life in adults with type 1 diabetes: further findings from the DIAMOND randomized clinical trial. Diabetes Care.

[11] Battelino T., Danne T., Bergenstal R.M., Amiel S.A., Beck R., Biester T. (2019). Clinical targets for continuous glucose monitoring data interpretation: recommendations from the international consensus on time in range. Diabetes Care.

[12] Beck R.W., Bergenstal R.M., Riddlesworth T.D., Kollman C., Li Z., Brown A.S. (2019). Validation of time in range as an outcome measure for diabetes clinical trials. Diabetes Care.

[13] Gabbay M.A.L., Rodacki M., Calliari L.E., Vianna A.G.D., Krakauer M., Pinto M.S. (2020). Time in range: a new parameter to evaluate blood glucose control in patients with diabetes. Diabetol Metab Syndr.

[14] McAuley S.A., Lee M.H., Paldus B., Vogrin S., de Bock M.I., Abrahamet M.B. (2020). Six months of hybrid closed-loop versus manual insulin delivery with fingerprick blood glucose monitoring in adults with type 1 diabetes: a randomized, controlled trial. Diabetes Care.

[15] Punke M.A., Decker C., Wodack K., Reuter D.A., Kluge S. (2015). Continuous glucose monitoring on the ICU using a subcutaneous sensor. Med Klin Intensivmed Notfmed.

[16] Punke M.A., Decker C., Petzoldt M., Reuter D.A., Wodack K.H., Reichenspurner H. (2019). Head-to-head comparison of two continuous glucose monitoring systems on a cardio-surgical ICU. J Clin Monit Comput.

[17] Sadhu A.R., Serrano I.A., Xu J., Nisar T., Lucier J., Pandya A.R. (2020). Continuous glucose monitoring in critically ill patients with COVID-19: results of an emergent pilot study. J Diabetes Sci Technol.

[18] Agarwal S., Mathew J., Davis G.M., Shephardson A., Levine A., Louard R. (2021). Continuous glucose monitoring in the intensive care unit during the COVID-19 pandemic. Diabetes Care.

[19] Tingsarat W., Buranasupkajorn P., Khovidhunkit W., Boonchaya-Anant P., Laichuthai N. (2022). The accuracy of continuous glucose monitoring in the medical intensive care unit. J Diabetes Sci Technol.

[20] Chow K.W., Kelly D.J., Rieff M.C., Skala P.A., Kravets I., Charitou M.M. (2021). Outcomes and healthcare provider perceptions of real-time continuous glucose monitoring (rtCGM) in patients with diabetes and COVID-19 admitted to the ICU. J Diabetes Sci Technol.

[21] Faulds E.R., Boutsicaris A., Sumner L., Jones L., McNett M., Smetana K.S. (2021). Use of continuous glucose monitor in critically ill COVID-19 patients requiring insulin infusion: an observational study. J Clin Endocrinol Metab.

[22] Perez-Guzman M.C., Duggan E., Gibanica S., Cardona S., Corujo-Rodriguez A., Faloye A. (2021). Continuous glucose monitoring in the operating room and cardiac intensive care unit. Diabetes Care.

[23] Boeder S., Kobayashi E., Ramesh G., Serences B., Kulasa K., Majithia A.R. (2023). Accuracy and glycemic efficacy of continuous glucose monitors in critically ill COVID-19 patients: a retrospective study. J Diabetes Sci Technol.

[24] Friman O., Soltani N., Lind M., Zetterqvist P., Balintescu A, Perner A. (2024). Performance of subcutaneous continuous glucose monitoring in adult critically ill patients receiving vasopressor therapy. Diabetes Technol Ther.

[25] Voglova Hagerf B., Protus M., Nemetova L., Mraz M., Kieslichova E., Uchytilova E. (2024). Accuracy and feasibility of real-time continuous glucose monitoring in critically ill patients after abdominal surgery and solid organ transplantation. Diabetes Care.

[26] Faulds E.R., Hester J.C., Badakhshi Y., Miller J.D., Basil R.C., Chandra S. (2025). Continuous glucose monitoring in the intensive care unit: a multicenter, retrospective hospital-based analysis. J Diabetes Sci Technol.

[27] Sun M.T., Li I.C., Lin W.S., Lin G.M. (2021). Pros and cons of continuous glucose monitoring in the intensive care unit. World J Clin Cases.

[28] Altman D.G., Bland J.M. (1983). Measurement in medicine: the analysis of method comparison studies. The Statistician.

[29] Lin L.I. (1989). A concordance correlation coefficient to evaluate reproducibility. Biometrics.

[30] van Steen S.C., Rijkenberg S., Limpens J., van der Voort P.H., Hermanides J., DeVries J.H. (2017). The clinical benefits and accuracy of continuous glucose monitoring systems in critically ill Patients-A systematic scoping review. Sensors (Basel).

[31] Charlson M.E., Pompei P., Ales K.L., MacKenzie C.R. (1987). A new method of classifying prognostic comorbidity in longitudinal studies: development and validation. J Chronic Dis.

[32] Elixhauser A., Steiner C., Harris D.R., Coffey R.M. (1998). Comorbidity measures for use with administrative data. Med Care.

[33] Gilbert T., Neuburger J., Kraindler J., Keeble E., Smith P., Ariti C. (2018). Development and validation of a hospital frailty risk score focusing on older people in acute care settings using electronic hospital records: an observational study. Lancet.

[34] Knaus W.A., Draper E.A., Wagner D.P., Zimmerman J.E. (1986). An evaluation of outcome from intensive care in major medical centers. Ann Intern Med.

[35] Vincent J.L., Moreno R., Takala J., Willatts S., De Mendonca A., Bruininget H. (1996). The SOFA (Sepsis-related organ failure assessment) score to describe organ dysfunction/failure. On behalf of the working group on sepsis-related problems of the european society of intensive care medicine. Intensive Care Med.

[36] Beck R.W., Riddlesworth T., Ruedy K., Ahmann A., Bergenstal R., Haller S. (2017). Effect of continuous glucose monitoring on glycemic control in adults with type 1 diabetes using insulin injections: the DIAMOND randomized clinical trial. JAMA.

[37] Lind M., Polonsky W., Hirsch I.B., Heise T., Bolinder J., Dahlqvist S. (2017). Continuous glucose monitoring vs conventional therapy for glycemic control in adults with type 1 diabetes treated with multiple daily insulin injections: the GOLD randomized clinical trial. JAMA.

[38] Beck R.W., Riddlesworth T.D., Ruedy K., Ahmann A., Haller S., Kruger D. (2017). Continuous glucose monitoring versus usual care in patients with type 2 diabetes receiving multiple daily insulin injections: a randomized trial. Ann Intern Med.

[39] Honarmand K., Sirimaturos M., Hirshberg E.L., Bircher N.G., Agus M.S.D., Carpenter D.L. (2024). Society of critical care medicine guidelines on glycemic control for critically ill children and adults 2024. Crit Care Med.

[40] Gunst J., Debaveye Y., Guiza F., Dubois J., De Bruyn A., Dauwe D. (2023). Tight blood-glucose control without early parenteral nutrition in the ICU. N Engl J Med.

[41] Adigbli D., Li Y., Hammond N., Chatoor R., Devaux A.G., Li Q. (2024). A patient-level meta-analysis of intensive glucose control in critically ill adults. NEJM Evid.

[42] The NICE-SUGAR Study Investigators (2012). Hypoglycemia and risk of death in critically ill patients. N Engl J Med.

[43] Heinemann L. (2022). Interferences with CGM systems: practical relevance?. J Diabetes Sci Technol.

[44] Hicks P., Huckson S., Fenney E., Leggett I., Pilcher D., Litton E. (2019). The financial cost of intensive care in Australia: a multicentre registry study. Med J Aust.

[45] Turchin A., Matheny M.E., Shubina M., Scanlon J.V., Greenwood B., Pendergrass M.L. (2009). Hypoglycemia and clinical outcomes in patients with diabetes hospitalized in the general ward. Diabetes Care.

