# Supplementary material for: Accuracy of continuous glucose monitoring in critically ill patients
Source: Crit Care Resusc. 2025 Oct 17;27(4):100118. doi: 10.1016/j.ccrj.2025.100118 (PMC12554099; doi:10.1016/j.ccrj.2025.100118)
Supplement: Multimedia component 1 [file mmc1.docx]

**Online Supplemental Material**

**Title**

Accuracy of Continuous Tissue Glucose Monitoring in Critically Ill Patients

**Authors**

John Santamaria

Ebony Selers

David Reid

**Contents**

e-Figure 1. Glucose Management Protocol

e-Table 1. Example download of data from the Dexcom Receiver

e-Table 2. Demographic and clinical details of patients, stratified by pre-existing diabetes

e-Table 3 Primary diagnoses based on the ANZICS modification of the APACHE III diagnostic codes

e-Table 4. Charlson, Elixhauser and Hospital Frailty Risk Scores for the 103 patients

e-Figure 2. Bland-Altman Plot, by Sensor type

e-Figure 3 Bland Alman limits of agreement plot, by Sensor type

e-Table 5. Hypoglycaemia rates in 5 trials of intensive or liberal glucose control

**e-Figure 1. Insulin Protocol**


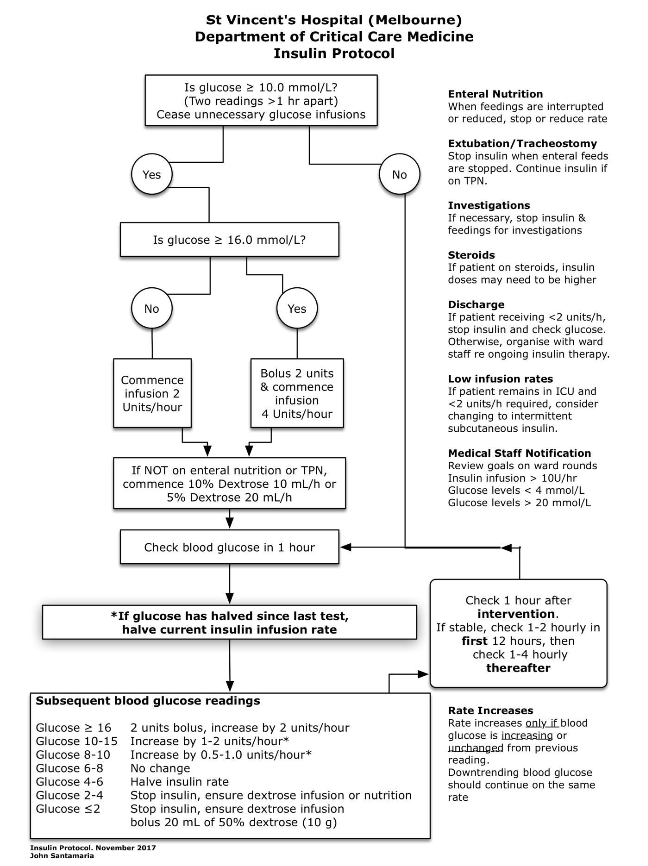


**e-Table 1**

A sample download from a Dexcom Receiver showing the 5-minute estimates of Tissue Glucose (EGV). The Blood/Capillary Glucose measurements (Calibration) are shown as well. The columns are sequence, date/time, sample (EGV or Calibration), estimated/measured glucose (mg/dL), Transmitter Time, and Transmitter Code.

Accuracy was assessed as the Tissue Glucose (EGV) minus Blood Glucose (Calibration) (e.g. 130-132) in the data listed here with these readings referenced are italicized, red colour and starred*.

While displayed in mg/dL, glucose measurements are displayed on the receiver in mmol/L.

| **538** | **2020-03-04T06:50:56** | **EGV** | **132** | **352673498** | **6PGSH** |
| --- | --- | --- | --- | --- | --- |
| **539** | **2020-03-04T06:55:56** | **EGV** | **126** | **352673798** | **6PGSH** |
| **540** | **2020-03-04T07:00:56** | **EGV** | **124** | **352674098** | **6PGSH** |
| **541** | **2020-03-04T07:05:56** | **EGV** | **124** | **352674398** | **6PGSH** |
| **542** | **2020-03-04T07:10:56** | **EGV** | **125** | **352674698** | **6PGSH** |
| **543** | **2020-03-04T07:15:56** | **EGV** | **126** | **352674998** | **6PGSH** |
| **544** | **2020-03-04T07:20:56** | **EGV** | **128** | **352675298** | **6PGSH** |
| ***545*** | ***2020-03-04T07:25:56*** | ***EGV*** | ***130*** | ***352675598*** | ***6PGSH**** |
| ***546*** | ***2020-03-04T07:26:25*** | ***Calibration*** | ***132*** |  | ***6PGSH**** |
| **547** | **2020-03-04T07:30:56** | **EGV** | **125** | **352675898** | **6PGSH** |
| **548** | **2020-03-04T07:35:56** | **EGV** | **125** | **352676198** | **6PGSH** |
| **549** | **2020-03-04T07:40:56** | **EGV** | **126** | **352676498** | **6PGSH** |
| **550** | **2020-03-04T07:45:56** | **EGV** | **128** | **352676798** | **6PGSH** |
| **551** | **2020-03-04T07:50:56** | **EGV** | **128** | **352677098** | **6PGSH** |
| **552** | **2020-03-04T07:55:56** | **EGV** | **127** | **352677398** | **6PGSH** |
| ***553*** | ***2020-03-04T07:56:34*** | ***Calibration*** | ***128*** |  | ***6PGSH**** |
| ***554*** | ***2020-03-04T08:00:56*** | ***EGV*** | ***125*** | ***352677698*** | ***6PGSH**** |
| **555** | **2020-03-04T08:05:56** | **EGV** | **126** | **352677998** | **6PGSH** |
| **556** | **2020-03-04T08:10:56** | **EGV** | **126** | **352678298** | **6PGSH** |
| **557** | **2020-03-04T08:15:56** | **EGV** | **126** | **352678598** | **6PGSH** |
| **558** | **2020-03-04T08:20:56** | **EGV** | **124** | **352678898** | **6PGSH** |

## **e-Table 2.** **Demographic and clinical details of patients, stratified by pre-existing diabetes.**

|  | All Patients | No History of Diabetes | Known History of Diabetes | *P*-value |
| --- | --- | --- | --- | --- |
| Number | 103 | 43 | 60 |  |
| Gender |  |  |  |  |
| Male | 72 (68) | 33 (46) | 39 (54) | 0.14 |
| Female | 31 (31) | 10 (32) | 21 (68) |  |
| Age, years | 63 (55-69) | 64.5 (52.5-69) | 62 (57-69) | 0.96 |
| Height, metres | 1.73 (1.67-1.80) | 1.74 (1.67-1.81) | 1.73 (1.64-1.80) | 0.60 |
| Weight, kg | 88 (79.0-109.7) | 86.8 (76.5-100.1) | 88.0 (79.0-109.0) | 0.87 |
| ICU origin  Emergency Department  Operating Room  General ward  Inter hospital transfer | 15 (14.6) 50 (48.5) 6 (10.7) 15 (26.2) | 4 (9.3) 22 (57.2) 5 (11.6) 12 (27.9) | 11 (18.3) 28 (46.7) 6 (10.0) 15 (25.0) | 0.67 |
| ICU Discharge location  Died  Ward  Other hospital  Home | 17 (16.5) 81 (78.6) 4 (8.9) 1 (1.0) | 11 (25.6) 30 (69.8) 2 (4.6) 0 | 6 (10.0) 51 (85.0) 2 (3.3) 1 (1.7) | 0.11 |
| Hospital Discharge location  Died  Home  Other hospital  Rehabilitation  Aged Care | 30 (29.4) 40 (39.2) 20 (19.6) 10 (9.8) 2 (2.0) | 13 (30.2) 14 (32.6) 11 (25.6) 4 (9.3) 1 (2.3) | 17 (28.8) 26 (44.1) 9 (15.3) 6 (10.2) 1 (1.7) | 0.68 |
| Diabetes | 60 (58.3) |  |  |  |
| Hypertension | 55 (53.4) | 19 (34.6) | 36 (53.4) | 0.08 |
| Ischaemic heart disease | 35 (34.0) | 9 (20.9) | 26 (43.3) | 0.02 |
| Chronic lung disease | 24 (23.3) | 14 (32.6) | 10 (16.7) | 0.10 |
| Current smoking | 13 (12.6) | 4 (9.3) | 9 (15.0) | 0.55 |
| 5 Common admission diagnoses | CABG (17) Sepsis (13) CABG+ (9) Arrest (6) Valve (6) | CABG+ (6) CABG (5) Cardiogenic (3) Pneumonia (5) Sepsis (4) | CABG (12) Sepsis (9) DKA (5) Cardiogenic (4) ICH (4) |  |
| Acute Physiology Score | 15 (11-21) | 15 (11-22) | 15.5 (11-19.5) | 0.92 |
| APACHE II | 20 (15-25) | 20 (15-27) | 20 (15-24) | 0.60 |
| Chronic Health  Normal  Chronic elective  Chronic emergency | 85 (82.5) 5 (4.9) 13 (12.6) | 34 (79.1) 2 (4.6) 7 (16.3) | 51 (55.0) 3 (5.0) 7 (10.0) | 0.61 |
| Maximum SOFA, 24H | 10 (7-12) | 10 (7-12) | 10 (7-12) | 0.78 |
| Mechanical ventilation | 100 (97.0) | 58 (96.7) | 56 (94.9) | 0.62 |
| Renal replacement therapy | 26 (25.2) | 10 (23.3) | 16 (26.7) | 0.44 |
| Enteral feeding | 84 (81.6) | 36 (83.7) | 48 (80.0) | 0.80 |
| Parenteral nutrition | 12 (11.7) | 8 (18.6) | 4 (6.7) | 0.12 |
| Inotropes | 95 (92.2) | 39 (90.7) | 56 (93.3) | 0.72 |
| Creatinine mmol/L | 0.10 (0.07-0.14) | 0.10 (0.07-0.14) | 0.11 (0.06-0.15) | 0.71 |
| eGFR (mL/min/1.73m^2^) | 62.2 (43.5-98.3) | 72.8 (44.5-100.3) | 59.8 (41.7-98.2) | 0.41 |
| Chronic kidney disease (eGFR<60) | 49 (47.6) | 19 (44.2) | 30 (50.0) | 0.96 |
| Bilirubin >20 mmol/L | 36 (35.0) | 20 (62.8) | 36 (60.0) | 0.06 |
| Base deficit < -5.0 | 63 (61.2) | 27 (62.8) | 36 (60.0) | 0.84 |
| Worst glucose 1^st^ 24H | 14.2 (12.5-16.4) | 13.0 (11.3-14.4) | 14.8 (13.4-17.4) | <0.001 |

## **e-Table 3. Primary diagnoses based on the ANZICS modification of the APACHE III diagnostic codes. ^1^**

| **Diagnostic Group** |  |
| --- | --- |
| 1207 – Postop Coronary bypass surgery | 16 |
| 503- Septic shock | 11 |
| 1212 – Postop Coronary bypass and valve Surgery | 10 |
| 1206 – Postop Cardiac valve surgery | 7 |
| 102 – Cardiac arrest | 6 |
| 1501 – Postop intracerebral haemorrhage | 6 |
| 702 - Diabetic ketoacidosis | 5 |
| 311 - Pancreatitis | 4 |
| 401 – Intracerebral haemorrhage | 4 |
| 408 – Other neurological disorder | 4 |
| 1503 – Postop subarachnoid haemorrhage | 4 |
| 211 – Other respiratory disorder | 3 |
| 213 – Viral pneumonia | 3 |
| 1401 – Postop Gastrointestinal perforation | 3 |
| 101 – Cardiogenic shock | 2 |
| 1506 – Postop Other neurological disorder | 2 |
| 103 – Aortic aneurysm | 1 |
| 201 – Aspiration pneumonia | 1 |
| 303 – Oesophageal varices | 1 |
| 308 – Gastrointestinal perforation | 1 |
| 402 – Subarachnoid haemorrhage | 1 |
| 407 – Seizure disorder | 1 |
| 501 – Sepsis without shock | 1 |
| 703 – Drug overdose | 1 |
| 901 – Renal failure | 1 |
| 1202 – Postop peripheral vascular disorder | 1 |
| 1211 – Aorto-bifemoral graft | 1 |
| 1409 – Postop GI fistula/abscess disorder | 1 |
| 1410 – Postop ischaemic bowl | 1 |

## **e-Table 4.** **Charlson, Elixhauser and Hospital Frailty Risk Scores for the 103 patients.**

| **Comorbidity** | **N=103** |
| --- | --- |
| Charlson Comorbidities ^2^ Myocardial infarction Cardiac failure Peripheral vascular disease Cerebrovascular disease Dementia Chronic pulmonary disease Connective tissue disorder Peptic ulcer disease Mild liver disease Diabetes, no complications Diabetes, complications Hemiplegia Renal Cancer Liver disease Metastatic cancer AIDS | 18 30 7 22 0 9 0 0 7 37 44 7 11 1 1 0 0 |
| Elixhauser Comorbidities ^3^ Congestive heart failure Cardiac arrhythmias Valve disease Pulmonary circulatory disorder Peripheral vascular disease Hypertension Paralysis Neurologic Chronic pulmonary disorder Diabetes, no complications Diabetes, complications Hypothyroid Renal failure Liver disease Peptic ulcer disease HIV Lymphoma Metastatic cancer Solid tumour Collagen vascular disease Coagulopathy Obesity Weight loss Fluids & Electrolytes Blood loss anaemia Deficiency anaemia Alcohol abuse Drug abuse Psychosis Depression | 29 36 20 8 6 31 7 11 9 23 52 2 11 9 0 0 1 0 1 1 10 0 24 30 2 4 12 4 3 2 |
| Charlson summation 1-2 >2 | 63 33 |
| Elixhauser summation 1-2 >2 | 32 70 |
| Hospital frailty risk score ^4^ >5 | 34 |

**References**

1. CORE A. ANZICS APD Data Dictionary. 2023; <https://www.anzics.com.au/wp-content/uploads/2021/03/ANZICS-APD-Dictionary-Version-6.1.pdf>. Accessed 20 April 2025.

2. Charlson ME, Pompei P, Ales KL, MacKenzie CR. A new method of classifying prognostic comorbidity in longitudinal studies: development and validation. *J Chronic Dis.* 1987;40(5):373-383.

3. Elixhauser A, Steiner C, Harris DR, Coffey RM. Comorbidity measures for use with administrative data. *Med Care.* 1998;36(1):8-27.

4. Gilbert T, Neuburger J, Kraindler J, et al. Development and validation of a Hospital Frailty Risk Score focusing on older people in acute care settings using electronic hospital records: an observational study. *Lancet.* 2018;391(10132):1775-1782.

**e-Figure 2.** **Bland Altman plots stratified by Sensor Type (G4, G6)**Tissue glucose minus the following blood glucose


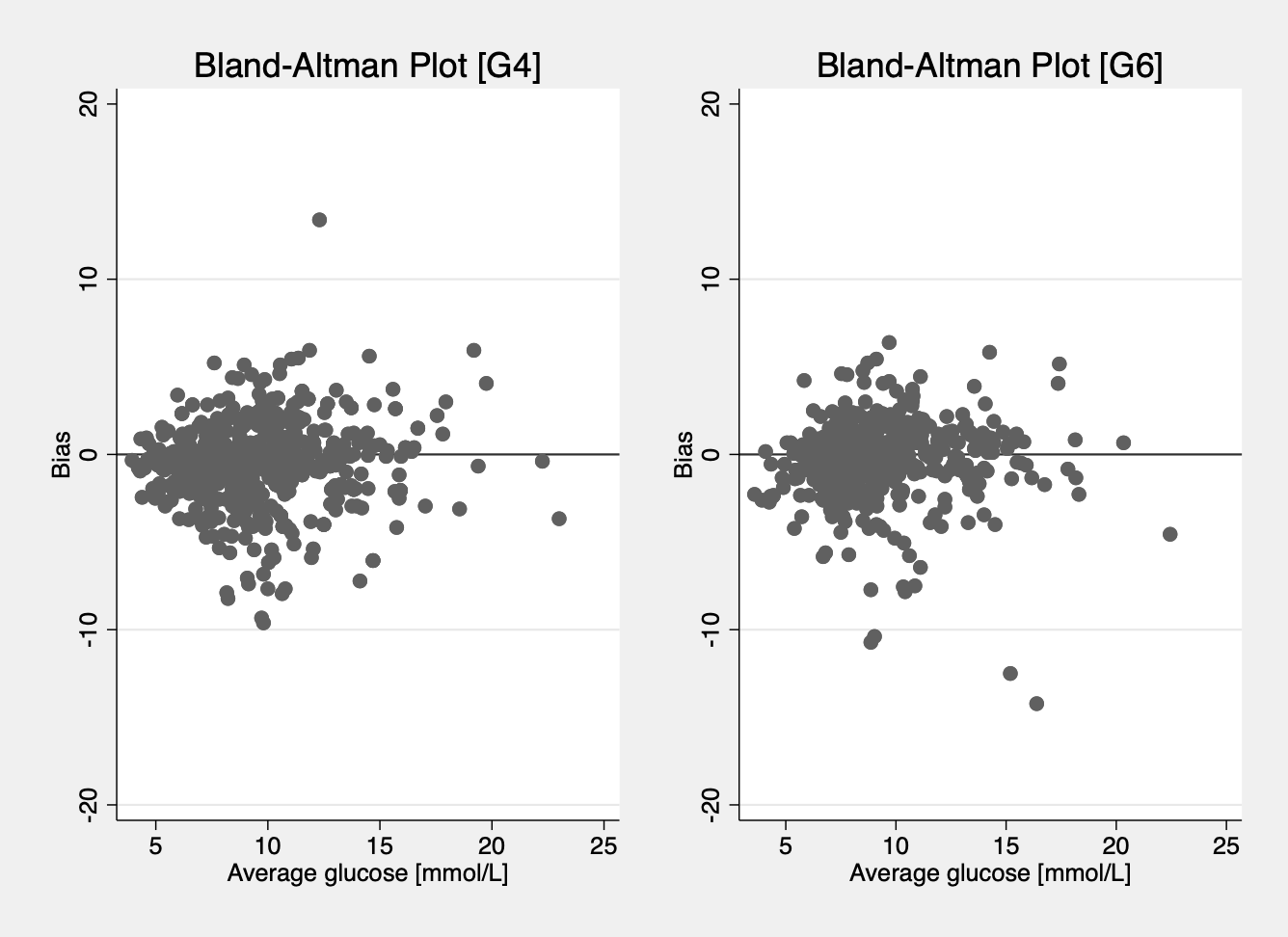


**e-Figure 3** **Bland Altman Limits of Agreement stratified by Sensor Type (G4, G6)**

**
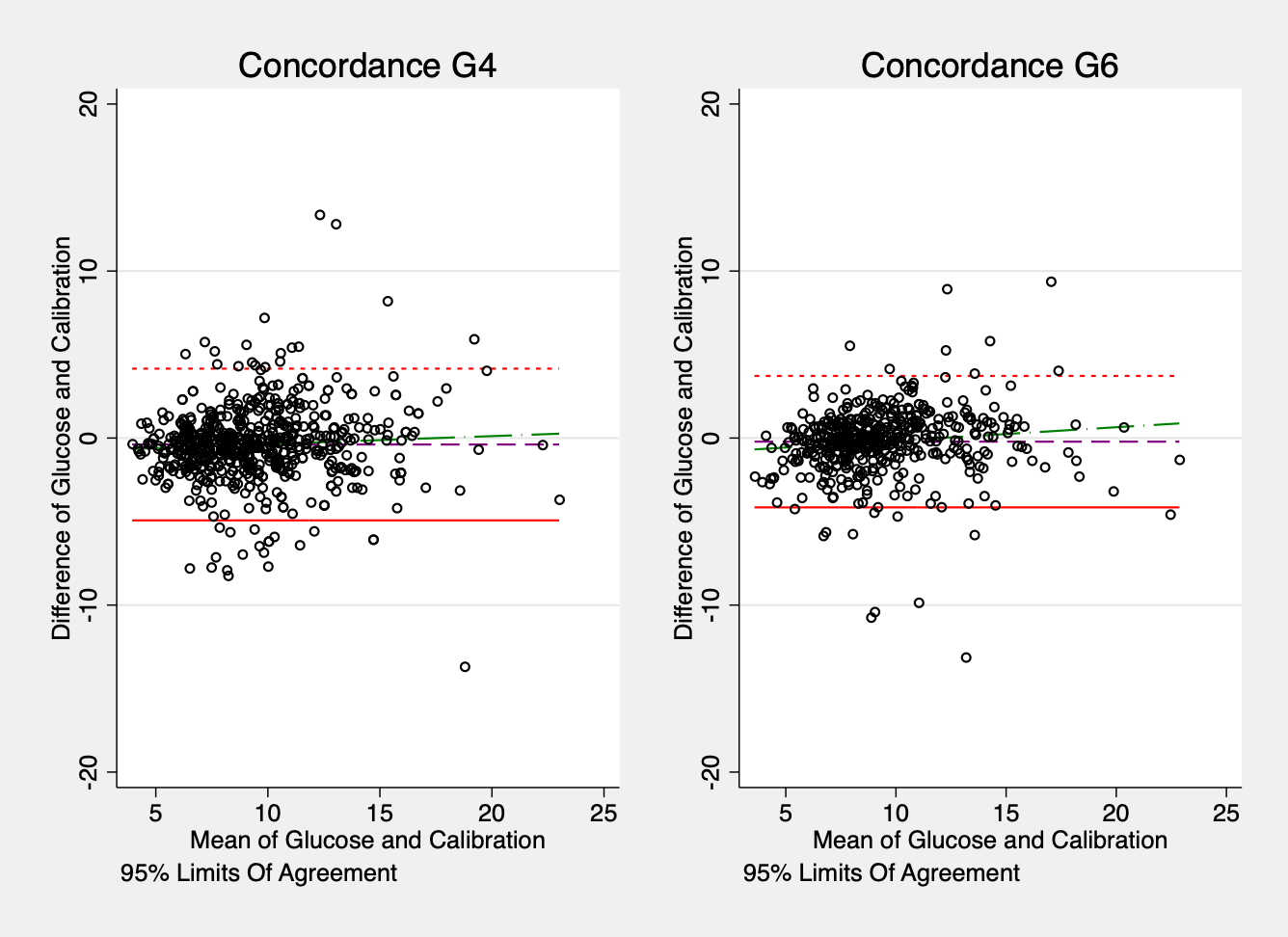
**

## **e-Table 5. Hypoglycaemia rates in 5 trials of intensive or liberal glucose control** Group refers to Control or Intensive glucose management except for Poole et al where the lower glucose threshold was classified as “comparator”. Target refers to the target ranges of the different groups. Total is the number of subjects in each group. Hypoglycaemia refers to the number of patients reported as having at least one hypoglycaemic event and % is the percentage of patients with at least one hypoglycaemic event.

|  | Van den Berghe ^5^ | | | Van den Berghe^6^ | | | NICE SUGAR^7^ | | | Poole^8^ | | | Gunst^9^ | |
| --- | --- | --- | --- | --- | --- | --- | --- | --- | --- | --- | --- | --- | --- | --- |
| Group | Control | Intense | Control | | Intense | Control | | Intense | Control | | Higher | Control | | Intense |
| Target | 10.0-11.1 | 4.4-6.1 | 10-11 | | 4.4-6.1 | <=10 | | 4.5-6.0 | 6-10 | | 10-14 | 10-11.9 | | 4.4-6.1 |
| Definition | <2.2 | <2.2 | <2.2 | | <2.2 | <2.2 | | <2.2 | <4.0 | | <4.0 | <2.2 | | <2.2 |
| Total | 783 | 765 | 605 | | 595 | 3014 | | 3016 | 209 | | 210 | 4622 | | 4608 |
| Hypoglycemia, number | 6 | 39 | 19 | | 111 | 15 | | 3016 | 38 | | 10 | 31 | | 47 |
| Hypoglycemia, % | 0.7% | 5.1% | 3.1% | | 18.7% | 0.5% | | 6.8% | 18.2% | | 5.7% | 0.7% | | 1.0% |

5. van den Berghe G, Wouters P, Weekers F, et al. Intensive insulin therapy in critically ill patients. *N Engl J Med.* 2001;345(19):1359-1367.

6. Van den Berghe G, Wilmer A, Hermans G, et al. Intensive insulin therapy in the medical ICU. *N Engl J Med.* 2006;354(5):449-461.

7. The NICE-SUGAR Study Investigators. Intensive versus conventional glucose control in critically ill patients. *N Engl J Med.* 2009;360(13):1283-1297.

8. Poole AP, Finnis ME, Anstey J, et al. The Effect of a Liberal Approach to Glucose Control in Critically Ill Patients with Type 2 Diabetes: A Multicenter, Parallel-Group, Open-Label Randomized Clinical Trial. *Am J Respir Crit Care Med.* 2022;206(7):874-882.

9. Gunst J, Debaveye Y, Guiza F, et al. Tight Blood-Glucose Control without Early Parenteral Nutrition in the ICU. *N Engl J Med.* 2023;389(13):1180-1190.

## 
